# A Feasibility Study of Photovoltaic—Electrolysis—PEM Hybrid System Integrated Into the Electric Grid System Over the Korean Peninsula

**DOI:** 10.3389/fchem.2021.732582

**Published:** 2021-09-14

**Authors:** Chang Ki Kim, Hyun-Seok Cho, Chang-Hee Kim, Wonchul Cho, Hyun-Goo Kim

**Affiliations:** ^1^ New and Renewable Energy Resource Map Laboratory, Korea Institute of Energy Research, Daejeon, South Korea; ^2^ Hydrogen Research Department, Korea Institute of Energy Research, Daejeon, South Korea

**Keywords:** solar irradiance, photovoltaic, electrolysis, proton exchange membrane, performance model

## Abstract

A photovoltaic–electrolysis–PEM hybrid model was developed for a feasibility study, and simulations of several scenarios in Korea were performed. The solar irradiance was derived from the University of Arizona solar irradiance based on satellite–Korea Institute of Energy Research model which provides the satellite imagery over the Korean peninsula every 15 min. In Korea, the annual average solar irradiance is 1,310 kWh m^−2^ with a maximum of 1,440 kWh m^−2^ in 2017. Electricity load and solar irradiance information were used to test the performance model of the photovoltaic–electrolysis–PEM hybrid system for baseload and several peak load shave runs. When the baseload was set at 4200 MW, the total capacity of the Photovoltaic plants was 58.5 GW_p_. In contrast, the hybrid system reduced the peak load more efficiently during daytime. In particular, the capacity factor of the Proton Exchange Membrane system increased in winter because the solar irradiance is relatively weak in that season. These results provide useful insights for the development of control logic models for the PV–electrolysis–PEM system in micro-grid setups.

## Introduction

Photovoltaic (PV) power plants are increasingly being installed all over the world to mitigate the impact of climate change (REN21, 2021). The extremely high capacity of PV power plants used to curtail the electricity penetration in the grid system. Furthermore, the intermittent characteristics of renewable energy makes it difficult for the system operator to control the electricity and frequency of voltage (Chen and Dominguez-Garcia, 2012). Therefore, a reserve power or back-up system is required for the PV power plants. A battery supply can be utilized for daily storage but it is not practical for a seasonal storage due to low energy density (Gupta et al., 2009). H_2_ gas produced by electrolysis is efficient as an energy carrier because it is converted into the electricity by using a fuel cell system. Therefore, an electrolysis–fuel cell coupled system is a good alternative to the conventional battery.

Several feasibility studies have been conducted on the use of PV–electrolysis–fuel cell system (e.g., Lehman and Chamberlin, 1991; Barbir, 2005b; Gibson and Kelly, 2008; Hwang et al., 2009; Gibson and Kelly, 2010; Paola et al., 2011; Joneydi Shariatzadeh et al., 2015; Özgirgin et al., 2015; Budak and Devrim, 2019). For example, Lehman and Chamberlin (1991) designed the PV–electrolysis–fuel cell system as a stand-alone system. A research series from Gibson and Kelly (2008; 2010) optimized the PV–electrolysis–fuel cell system to increase the H_2_ generation efficiency for a fuel cell vehicle. Hwang et al. (2009) performed case studies to verify the effectiveness of the fuel cell system coupled with the PV module for a stand-alone energy management system. More recently, PV–electrolysis–fuel cell system is extended to a cogeneration system for heat and electricity (Özgirgin et al., 2015). Budak and Devrim (2019) designed methanol and water electrolyzers for a 1.2 kW fuel cell system demand which fulfilled the energy requirement of a household. When compared with a water electrolyzer, the methanol electrolyzer can produce 27% more H_2_ gas. The above mentioned researches were demonstrated as a stand-alone or off-grid system, however, Dahbi et al. (2018) attempted to integrate the PV–fuel cell hybrid system into the grid for power management. They optimized the design and algorithm of a hybrid system in the laboratory scale. Ghenai and Bettayeb (2019) attempted to design a grid connected renewable energy system to fulfil the electric load of the building with high penetration of renewable energy, low greenhouse gas emissions, and low cost of energy on the University campus. In their research, the grid-tied solar PV–fuel cell hybrid power system exhibited a good performance for the tested system architectures. Ebaid et al. (2015) employed the H_2_ gas turbine instead of the fuel cell system as the electricity generator. They analyzed the PV–H_2_ gas turbine system to generate a baseload of 100 MW.

Since the Renewable Energy 3,020 policy was announced in 2017, Korea has deployed renewable energy systems. The main objective of the policy is to install 48 GW PV power plants by 2030 to increase the penetration rate of renewable energy to 20%. In this situation, a high ramp rate for the PV power generation due to fast moving clouds causes an unstable voltage frequency in the short term. Further, a huge curtailment or duck-curve phenomenon expected. Thus, the purpose of this study is to design a PV–electrolysis–fuel cell hybrid model and test its performance for a given electricity demand or load within a grid system. The remainder of this article is organized as follows: *Current Status of Renewable Energy in Korea* introduces the current status of renewable energy in Korea. *Numerical Simulation Design* briefly describes the design for the numerical simulation modeling. Subsequently, the evaluation of the data is presented with short discussions in *Results and Discussions*. Finally, *Conclusion* summarizes the major findings of this study.

## Current Status of Renewable Energy in Korea

### Annual Solar Irradiance

**FIGURE 1:**
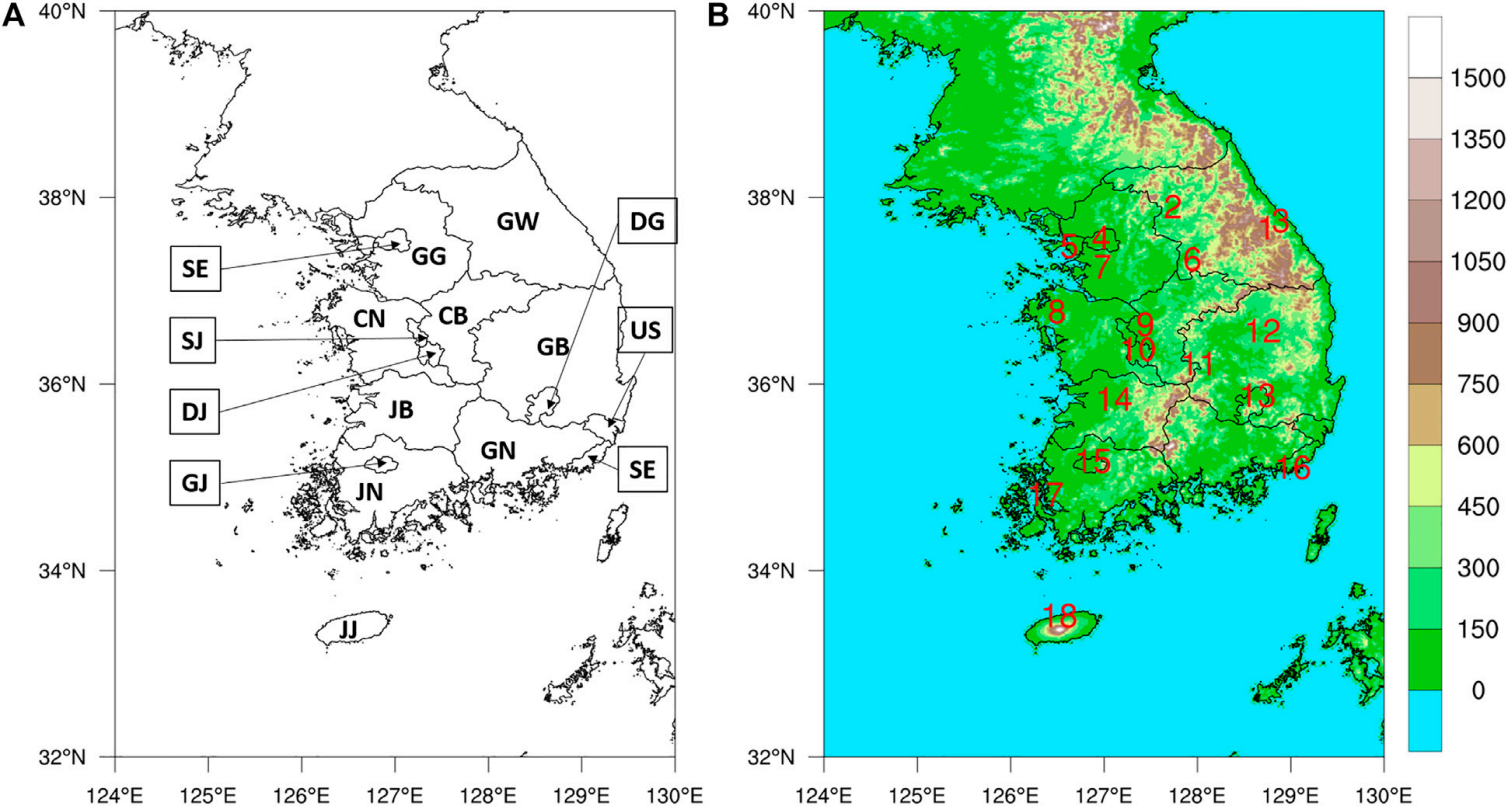
Administrative division in Korea **(A)** and Topography **(B)** in the UASIBS–KIER model domain.

**FIGURE 2:**
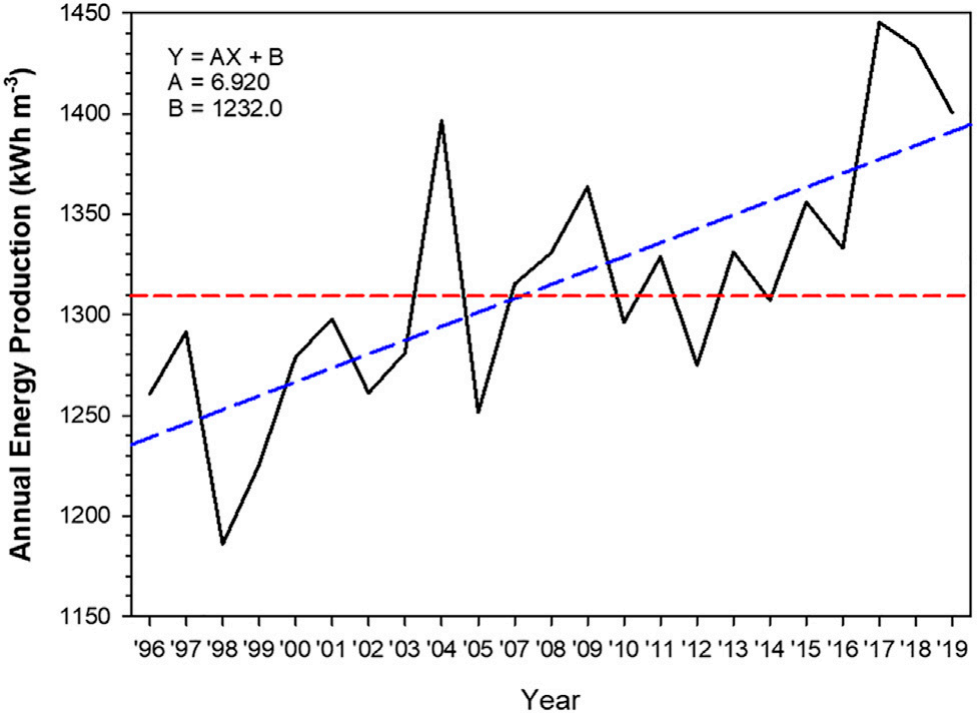
Annual energy production as a function of year that is derived from the solar irradiance. Blue dashed line indicates the regression line from 1996 to 2019. Red dashed line is the climatological mean from 1996 to 2019.

**FIGURE 3:**
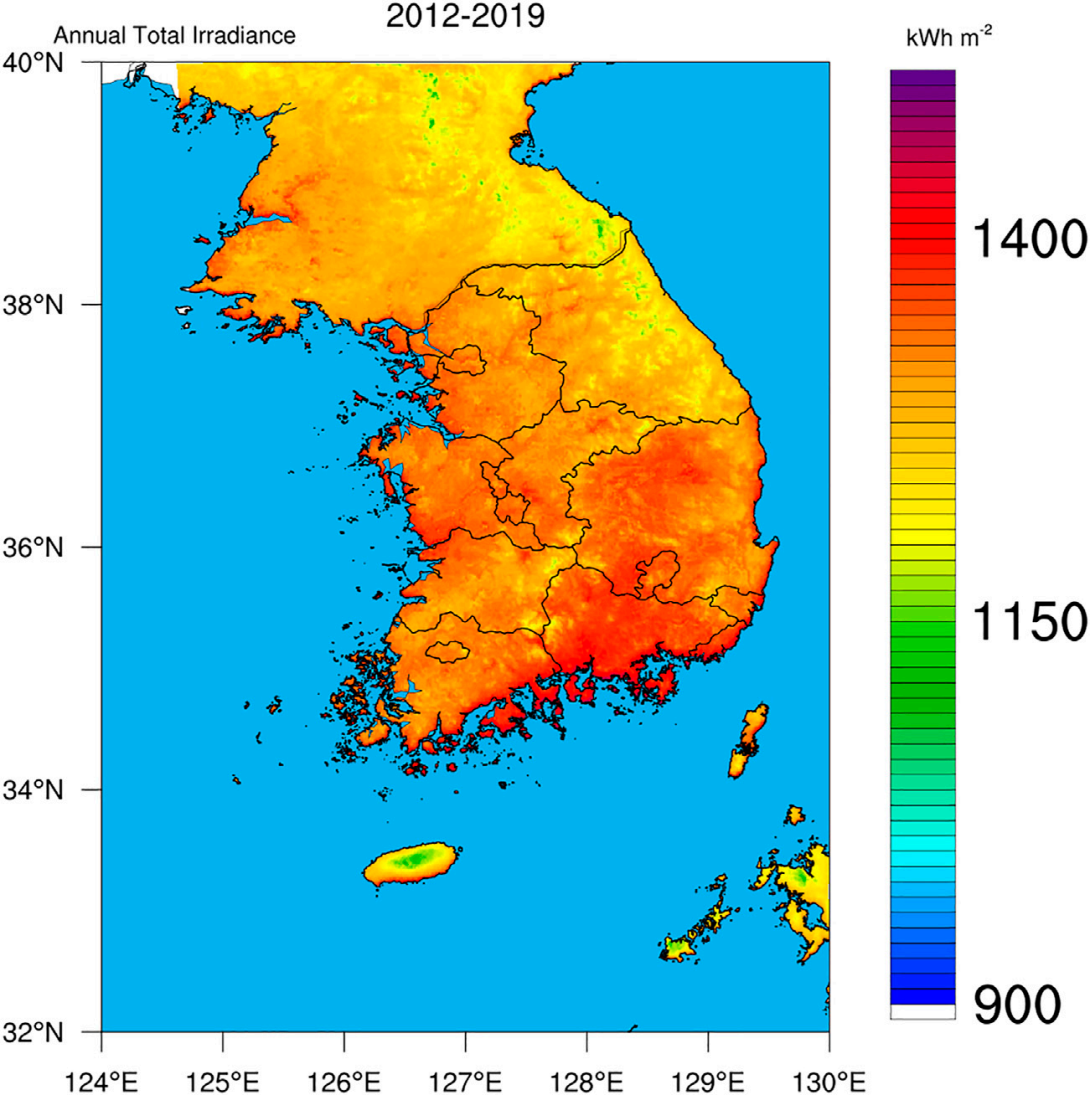
Horizontal distribution of annual total irradiance from 2012 to 2019.

The University of Arizona Solar Irradiance Based on Satellite–Korea Institute of Energy Research (UASIBS–KIER) model estimates the solar irradiance in a 1 km × 1 km grid cell over the Korean Peninsula (32° N–40° N, 124° E–130° E) using satellite imagery (Kim et al., 2017). Figure 1 shows the target area where solar irradiance is available. Annual solar irradiance estimated from 1996 to 2019 is given in Figure 2, which implies that the solar power generation can be gradually increased in Korea using enhanced solar irradiance. The average value of annual solar irradiance for 24 years is 1,310 kWh m^−2^. In 2017, the highest value of solar irradiance was recorded which exceeded 1,400 kWh m^−2^. During the brightening span from 2012 to 2019, the spatial distribution of annual solar irradiance indicates that southern part of the Korean Peninsula is an appropriate region for solar power generation (Figure 3).

### Electricity Load in Korea

**FIGURE 4:**
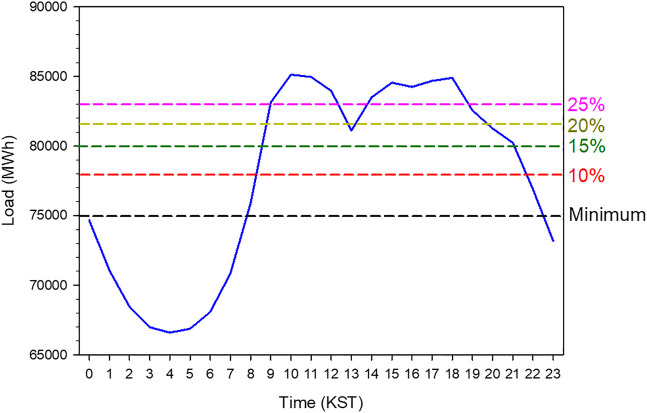
A time series of electricity load averaged over 2 years from 2017 to 2018 as a function of hour. The dashed line indicates the level for peak load shave.

**FIGURE 5:**
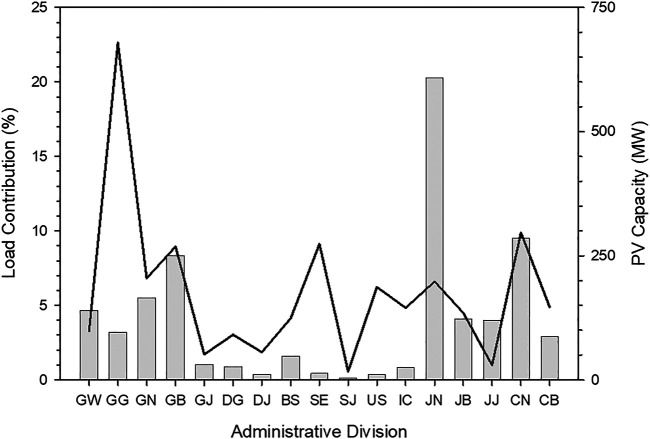
Regional contribution of electricity load to national electricity load **(solid line)** and installed capacity of PV power plants **(vertical bar)**.

Electricity load data is available in the Electric Power Statistics Information System (EPSIS), which is operated by the Korea Power Exchange (KPX). EPSIS data are hourly statistics but separate load data is not available for each administrative division in Korea. Figure 4 shows the electricity load in Korea as a function of time from 2016 to 2019. This load includes all sectors, which means that various sectors such as industrial and residence are combined. The electricity load behavior is consistent with solar irradiance, i.e., electricity demands increase when Sun rises and vice versa. In particular, electricity load continuously decreases until four Korean Standard Time (KST = Universal Time Coordinate +9 h) at which the lowest value is observed. During daytime, the load profile has two peaks, in the morning and afternoon. This can be attributed to the relative reduction of electricity usage during lunch time. Data on the hourly electricity load is not available for each administrative division but annual electricity load for each division is obtained from the EPSIS data. The regional contribution of load to national scale is shown in Figure 5. The electricity demand is the maximum in GG division. With the regional contribution of electricity load, the installed capacity of the PV plants in 2019 is presented in Figure 5. PV power plants of more than 700 MW capacity have been installed in JN division. Therefore, this study uses the JN division as the investigation region.

## Numerical Simulation Design

### Performance Modeling

**FIGURE 6:**
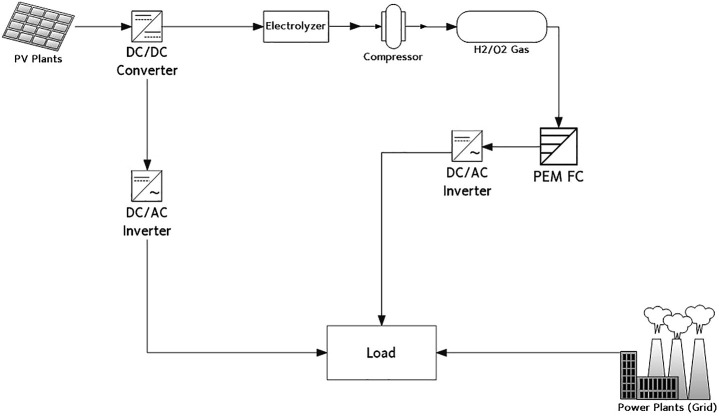
A block diagram for PV–Electrolysis–PEM hybrid model.

As mentioned in the previous section, local or regional electricity load data is not available and therefore it is not easy to simulate the microgrid system for the PV and electrolysis system. However, for the monthly or annual energy production, we can estimate the capacity of solar power plants in combination with water electrolysis where the fuel cell system is used as the reserve power. Figure 6 shows the block diagram employed in this study. The electric power in direct current is derived from the solar irradiance and nameplate efficiency at standard test conditions. Subsequently, the direct current from PV module is supplied by two methods. The priority is to supply the electricity to the grid through an inverter but excess electricity is needed to operate the electrolyzer for the production of H_2_ and O_2_. H_2_ gas, stored in the gas tank can be converted into electricity using the fuel cell system which is connected to the grid. However, this is not a management or control model for determining if the generated solar power is connected to the grid or water electrolysis system. When comparing the electricity load, the power in excess to the threshold value can be transmitted to the water electrolyzer.

### Photovoltaic–Electrolysis–Proton Exchange Membrane System

The major components of the performance model are PV, water electrolysis, and fuel cell system. The generated solar power in direct current (*P*_*DC*_) is formulated as follows:

$$P_{DC}=\frac{I_{POA}}{I_{o}}\times P_{\max}\times \left(TF\right)\times \left(SF\right),$$ (1)

where, *I*_*POA*_, *I*_*o*_, and *P*_max_ denote the plane of array (POA) irradiance, reference irradiance at standard test condition, and peak power (i.e., capacity). At the standard test condition, usually the reference irradiance is defined as 1000 W m^−2^ at 25°C and 1 atm. TF and SF are the loss factors due to cell temperature and soiling on the cell surface, respectively. Dobos (2014) estimated the TF using linear regression of the cell temperature with coefficients that are directly taken from the individual PV module specifications. The POA irradiance indicates the solar irradiance incident to the normal surface of the solar panel and its formulation is also dependent on the installation characteristics of PV plants, such as the slope and azimuth angles for solar panels. However, the information on these characteristics is unavailable. Instead, this study assumes that the slope and azimuth angles correspond to the latitude and 180°, respectively. The dependence of cell temperature is ignored and therefore, we set *P*_max_ to 700 MW_p_ based on the EPSIS data. Meanwhile, the generated solar power is transmitted into the grid to balance the load. To connect to the grid system, the direct current is converted into alternating current by the inverter. This study assumes that the efficiency of the inverter (*η*_*inv*_) is 90%, based on Dobos (2014).

Proton Exchange Membrane (PEM) electrolysis and fuel cell system are employed for the hydrogen production and reserve power generation. The 2 MW PEM electrolyzer stack and 100 kW PEM fuel cells consists of a 1,250 cm^2^ active area of 200 cells are considered as unit module for constructing the 700 MW PEM electrolyzer-fuel cells co-generation system. In this study, the PEM electrolyzer and fuel cells system efficiency are assumed to be 75% (LHV, 44.0 kWh/kg) and 65% (LHV, 12.8 kWh/kg) respectively. Note that the electrolyzer’s efficiency is the ratio equals the ideal power that is thermodynamically required for a certain hydrogen production rate divided by the electrical power for it expended. The definition of electrolysis efficiency is based on the following assumptions. 1) Electrolyzer operate at 20–80°C with only electricity as the energy source; 2) the heat of vaporization has to be provided by the electrolysis process; 3) the electricity which is applied to the electrolyzer’s system has to be equal to the enthalpy of water electrolysis reaction. Here we considered the case for converting hydrogen back to electricity (i.e., fuel cells), the chemical energy according to the Lower Heating Value (LHV) is used in the conversion steps for the evaluation of the overall energy efficiency of the complete conversion process from PV to PEM electrolyzer and fuel cells. The overall electrolyzer’s efficiency is defined as the product of the voltage (*η*_*voltage*_) and the current efficiency (*η*_*current*_) as below (Smolinka et al., 2015)

$$\eta_{system}=\eta_{stack}\times \left(\eta_{voltage}\times \eta_{current}\right)\times \eta_{BOP}$$ (2)

with *η*_BOP_, the efficiency for the balance of plant that is assumed to be 95% in this study. The electrical power consumed by the stack needed to produce hydrogen is formulated by the relationship between voltage and current in stack, i.e., $P_{stack}=I_{stack}\times V_{stack}$.

$$\eta_{voltage}=\frac{\left(\Delta H_{R}^{O}-\Delta H_{V}^{O}\right)\times {\dot{n}}_{H_{2}}}{P_{stack}}=\frac{LHV\times {\dot{n}}_{H_{2}}}{P_{stack}},$$ (3)

$$\eta_{current}=\frac{{\dot{n}}_{H_{2,}\text{Real}}}{{\dot{n}}_{H_{2,}\text{Ideal}}}=\frac{{\dot{n}}_{H_{2,}\text{Real}}}{I\times {\left(n\times F\right)}^{-1}},$$ (4)

where the *ṅ*_H2_ is the molar amount of produced hydrogen (mol s^−1 m−2^) theoretically by Faraday’s law, F is the Faraday constant (C mol^−1^), n is the number of electron participating in the reaction, I is the current (A)

The fuel cell efficiency is defined as a ratio between the electricity produced and hydrogen consumed and the electricity produced is a product between voltage and current. Also, the hydrogen consumed is directly proportional to current and thus the molar flow rate for H_2_ into the PEM fuel cells (hydrogen consumption) at the inlet of the channel can be defined thus:

$${\dot{n}}_{H_{2}}=N_{cell}\times s\times \frac{1}{2F},$$ (5)

where s (=1.2) is the stoichiometry of H_2_, and N_cell_ is the number of cells in the PEM fuel cells.

We assumed that the hydrogen is supplied to the cell in excess of that required for the reaction stoichiometry. The excess can be recirculated back into the stack so it does not change the fuel cell efficiency. The reverse of the stoichiometry is fuel utilization, the ratio between the amount of hydrogen actually supplied to the cells and that consumed in the electrochemical reaction. Here we chose the stoichiometry number 1.2 which represents the 88% of fuel utilization (Barbir, 2005a)

### Scenarios for Performance Model

In the present study, simulations of the performance model for two consecutive years (2017–2018) are carried out, because the annual solar irradiance in the last 2 decades was the maximum in these 2 years (Figure 2). Solar irradiance derived from the grid data of the UASIBS–KIER model using satellite imagery is averaged over the investigation region, i.e., the JN division, to generate the time series of solar power generation using Eq. 1. The reference run is done on the baseload in which the PV–electrolysis–PEM system supplies electricity for 24 h at a constant electricity load. In the baseload run, the solar power generation is employed to supply electricity into the grid and electrolysis during the daytime. During cloudy or rainy weather, the reduced solar power generation can be compensated for by the electricity produced by the PEM system with H_2_ storage. During the night, a reserve power system which includes the PEM system with H_2_ storage supplies the electricity into the grid to fulfill the electricity load. The PV power (*P*) is calculated using the following two equations at a given electricity load (*L*):

$$\sum_{i=1}^{24}\left(L_{i}\right)=\sum_{i=H_{r}}^{H_{s}}\left(P_{i}\right)+\sum_{i=H_{s}}^{H_{r}}\left(F_{i}\right)\times \eta_{inv},$$ (6)

$$\sum_{i=H_{s}}^{H_{r}}\left(F_{i}\right)\times \eta_{inv}=\sum_{i=H_{r}}^{H_{s}}\left(P_{i}-L_{i}\right)\times \frac{\eta_{PEM}}{\eta_{H_{2}}},$$ (7)

where F, *η*_*PEM*_, and *η*_*H2*_ are the PEM power, and the efficiency of water electrolysis and PEM system, respectively (Budak and Devrim, 2019). The information on efficiency is listed in Table 1. In the performance model, the PV system is operational only during the daytime from *H*_*r*_ (sunrise) to *H*_*s*_ (sunset). Using *p*, *η*_*inv*_, and Eq. 1, *P*_max_, i.e., the total capacity can be derived for the baseload operation.

**TABLE 1:** Summary of PV–Electrolysis–PEM system components.

Model	Parameter	Value
PV	Capacity (P_max_)	700 MW_p_
	Azimuth Angle	180
	Slope Angle	Latitude
	Inverter Efficiency (η_inv_)	0.90
PEM	PEM Efficiency (η_PEM_)	1.2 kWh m^−3^
Electrolysis	Electrolysis Efficiency ((η_H2_)	4.4 kWh m^−3^

The other scenarios are related to the peak load shave model, which means that a curtailment at peak load is prescribed and subsequently the excess solar power generated is transmitted to the water electrolysis system to produce H_2_ and O_2_ gas. Figure 4 shows an example of the daytime load for two consecutive years in the JN division. S1 is the simulation in which the peak load is reduced to the lowest quartile (25%) of the daytime load (see the pink dashed line in Figure 4). In other words, it is identical to the baseload and must be increased up to the lowest quartile of the daytime load. As shown in Figure 4, the elevated baseload level during daytime might result in the excessive load at night which is usually disposed. Consequently, the best method is to completely offset the peak or daytime load by using only the PV–electrolysis–PEM system (see the minimum level in Figure 4). Moreover, this study investigates the capability of the installed PV system to operate the electrolysis system with the PEM generator level where the shaved peak load is reduced from 25 to 15% in the simulations (S2 and S3). Additionally, four simulations (S4 to S7) are performed in the feasibility study to predict the installed PV capacity to accomplish the peak load shave. S4 and S5 increase the PV capacity by 50% of the current total capacity of PV plants in the JN Division. However, the level for the peak load shave is different for the two simulations; 15% in S4 run and 10% in S5 run. In S6 and S7 simulations, the prescribed PV capacity is twice the currently installed capacity of the PV system. Table 2 summarizes the simulation scenarios.

**TABLE 2:** The design for numerical simulation. B1 run is the baseload run and therefore PV capacity and the level for peak load shave are not determined in the simulation.

Simulation	Baseload	PV Capacity	Level for Peak Load Shave
B1	4200 MW	-	-
S1	125%	700 MW_p_	25%
S2	120%	700 MW_p_	20%
S3	115%	700 MW_p_	15%
S4	115%	1,050 MW_p_	15%
S5	110%	1,050 MW_p_	10%
S6	110%	1,400 MW_p_	10%
S7	110%	1750 MW_p_	10%

## Results and Discussions

**FIGURE 7:**
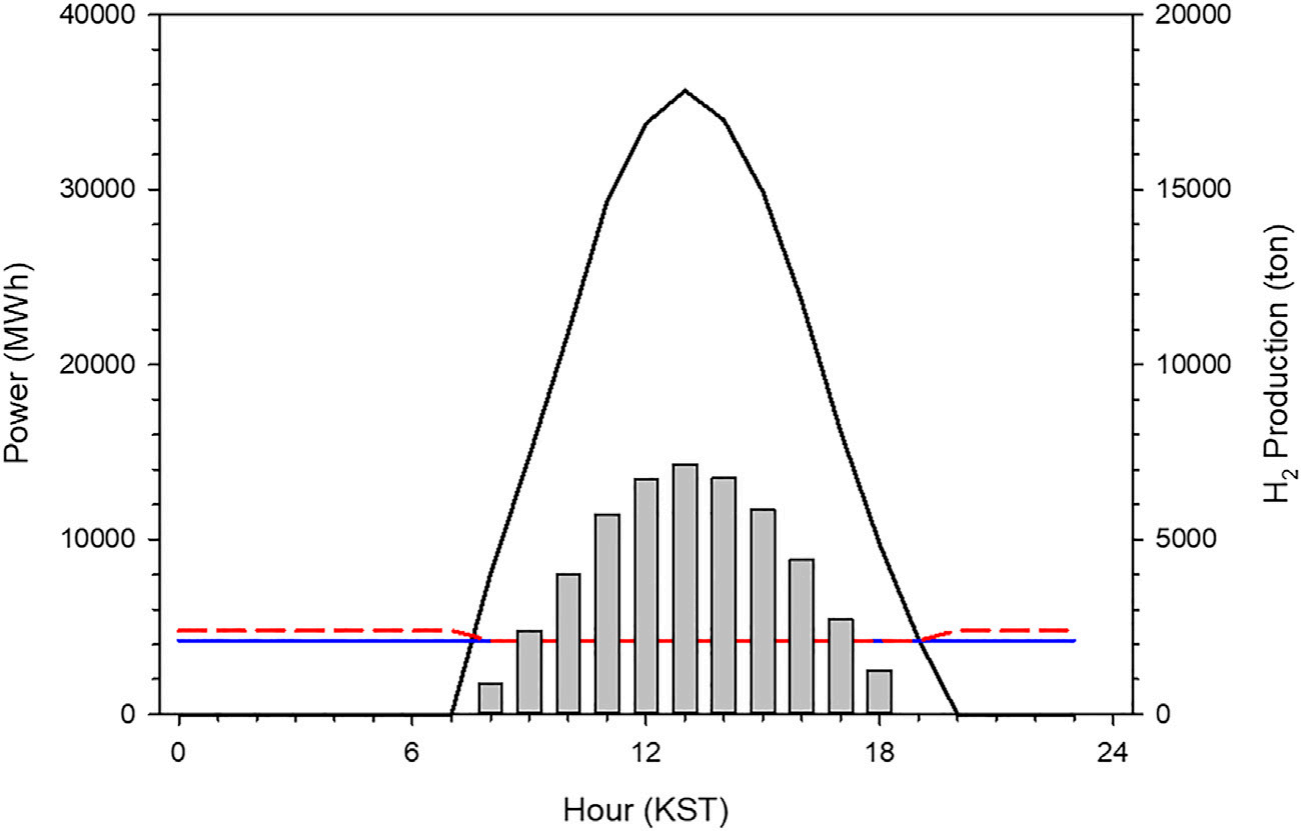
A time series of solar power generation transmitted into electrolysis **(black solid line)**, baseload **(blue solid line)** and electricity by generated by PEM and PV power plants **(red dashed line)** that come from the B1 run. Vertical bar means the production of H_2_ gas by the electrolysis.

The results from the baseload simulation performed indicates that the PV capacity is larger than 58.5 GW_p_ to supply the electricity at a rate of 4,200 MWh in the JN division for 24 h by employing the PV–electrolysis–PEM model. Figure 7 shows the result from the baseload run that is averaged over the 2 years from 2017 to 2018. Solar power generation dominates the electricity generation during the daytime. Compared to the electricity demand of 4,200 MWh, the excess power which is approximately 210 GWh is accumulated between 8 and 19 KST. The excess electricity is the power source for producing H_2_ gas through the water electrolysis system. Therefore, the production of H_2_ gas is proportional to the surplus electricity. On an average, the H_2_ productivity is the maximum at noon. H_2_ gas produced by the excessive solar energy can be employed to supply electricity at night. The electric power from the PEM system is greater than the baseload; 4,788 MWh power is generated by the reserve system but the demand is only 4,200 MWh. Thus, the PV–electrolysis–PEM system controls the baseload demand like charcoal power plants. Nevertheless, the total capacity is significantly large to be installed due to a practical economical problem.

**FIGURE 8:**
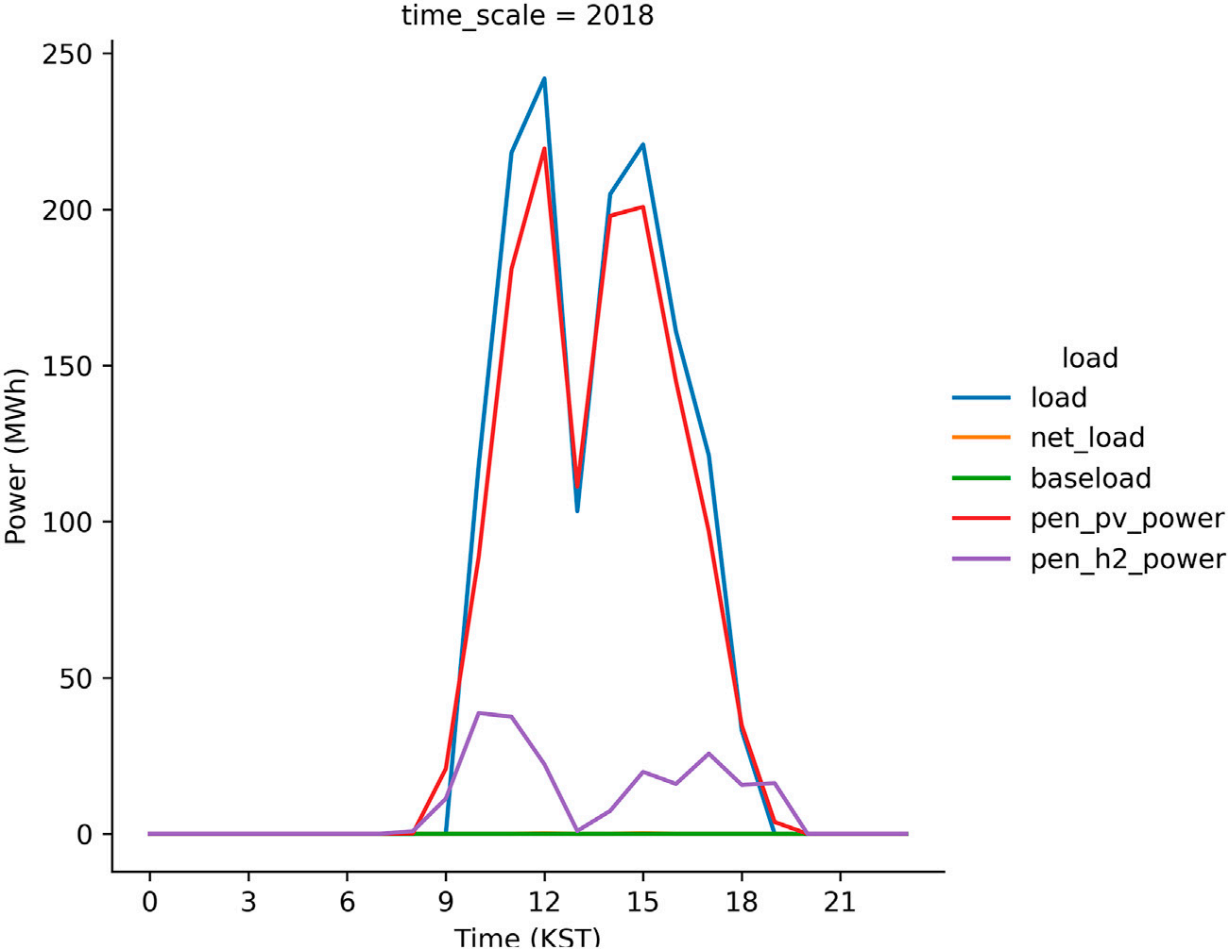
A time series of peak load **(blue line)**, solar power generation connected into grid **(red line)** and electricity generation due to PEM system **(purple line)** from the S1 run in 2018. *Y*-axis in the figure is offset by the baseload **(green line)** to highlight the peak load during daytime.

**FIGURE 9:**
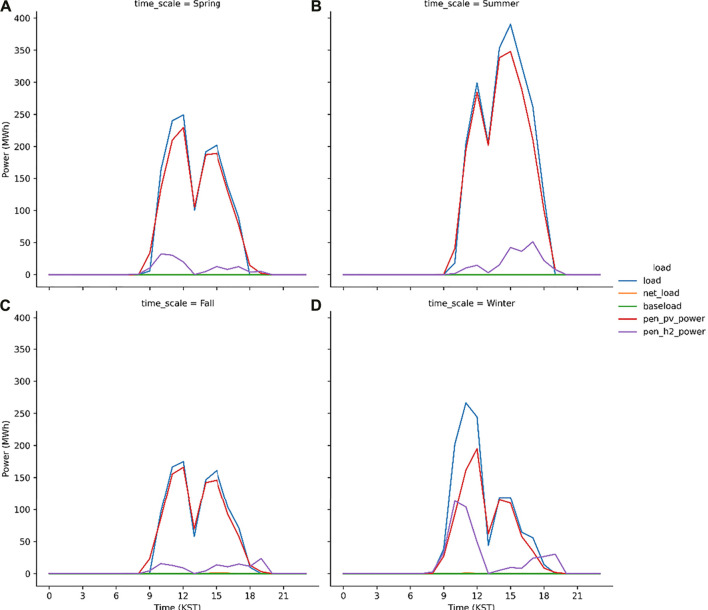
Same as Figure 8 but for spring **(A)**, summer **(B)**, fall **(C)** and winter **(D)**.

**FIGURE 10:**
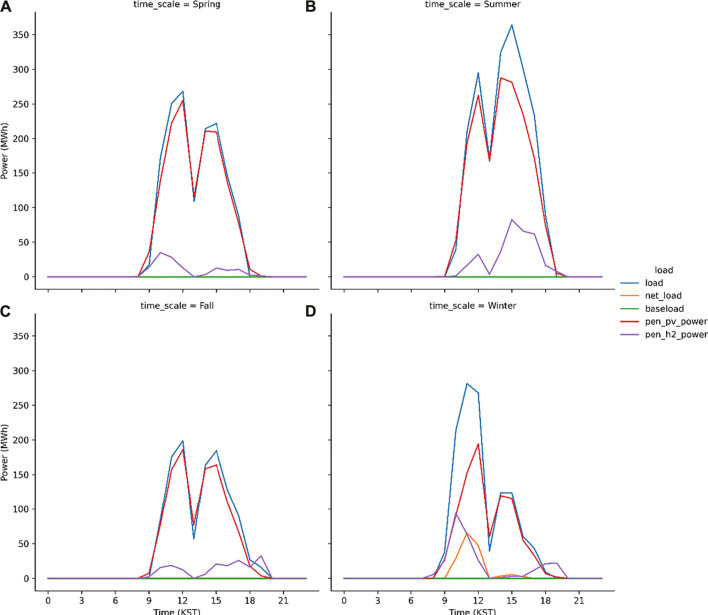
Same as Figure 9 but in 2017.

The discussed hybrid model works better as the peak load shave model which is proved from the S1 run (Figure 8). The peak load (= total load—baseload) at noon is 250 MWh when the PV power generation is 230 MWh. The difference between the peak load and PV power generation is 20 MWh that is compensated by the PEM system which generates the electric power using H_2_ gas. The seasonal characteristics are evident in Figure 9. In spring and fall, the capacity factor of the PEM system is expected to be relatively lower than the other seasons. This is because that sky is usually clear in these seasons in Korea (Chung et al., 2004; Ho et al., 2006; Ha et al., 2012) and therefore, the electrolysis system operates to produce H_2_ gas. However, in winters, the PEM system generates electricity in the morning, i.e., the 100 MWh electricity power is supplied to the grid or load at 10 KST. In the present study, the PV–electrolysis–PEM model simulations are performed for each scenario during two consecutive years and subsequently the results are considered from the simulation in the second year. In the first year, the electricity demand is not compensated for by the PV plants and PEM system in the first winter season, i.e., January to February 2017 because the model considers an empty H_2_ gas storage during the first year. Figure 10 illustrates the seasonal variations in the simulated load and power in 2017. Except the winter season, the trend is very similar to the simulation results in the second year (Figure 9). The PV and PEM power systems cannot supply the electricity demand in winter to make a positive net load in the morning hours. The load characteristics during the second-year changes, as evident from the simulation results. This is because the H_2_ gas accumulated in 2017 is sufficient to shave the peak load in winter.

**FIGURE 11:**
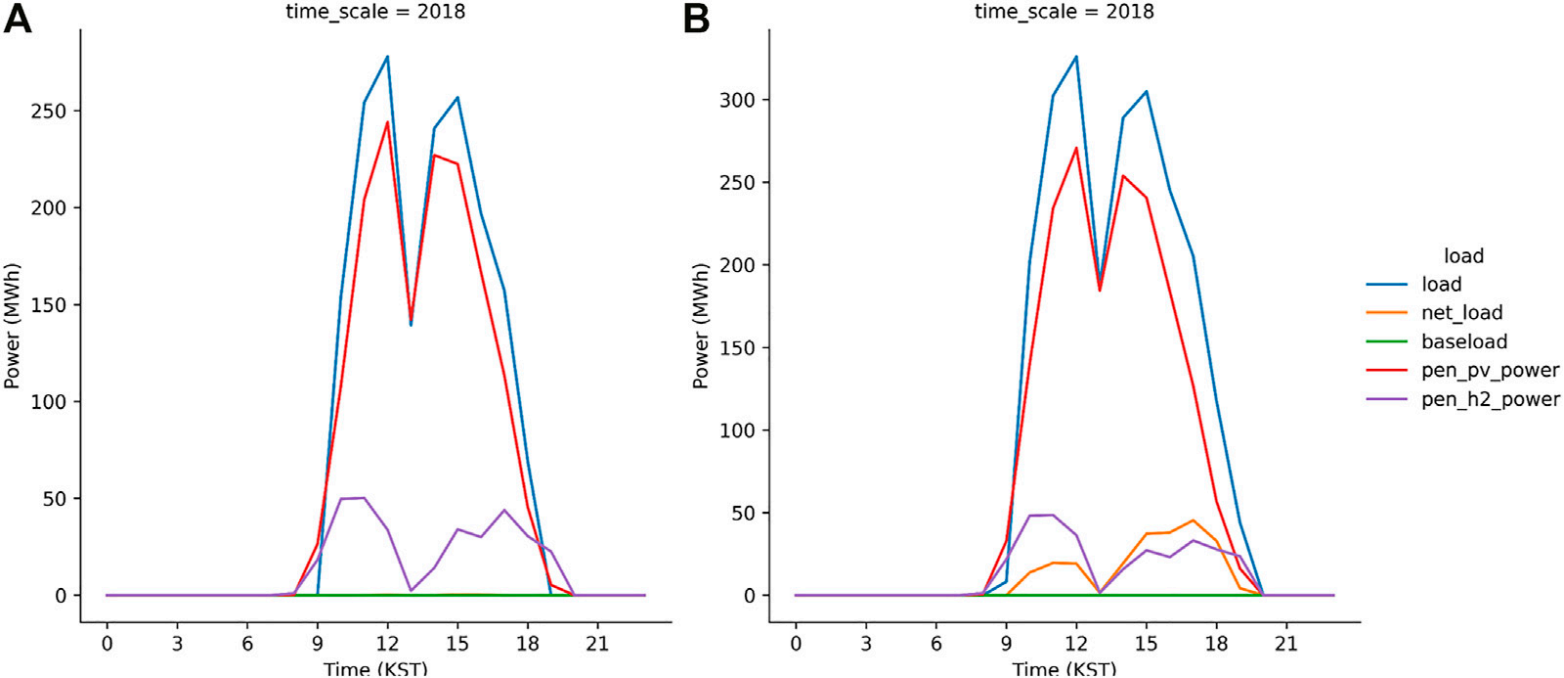
Same as Figure 8 but from S2 run **(A)** and S3 run **(B)**.

**FIGURE 12:**
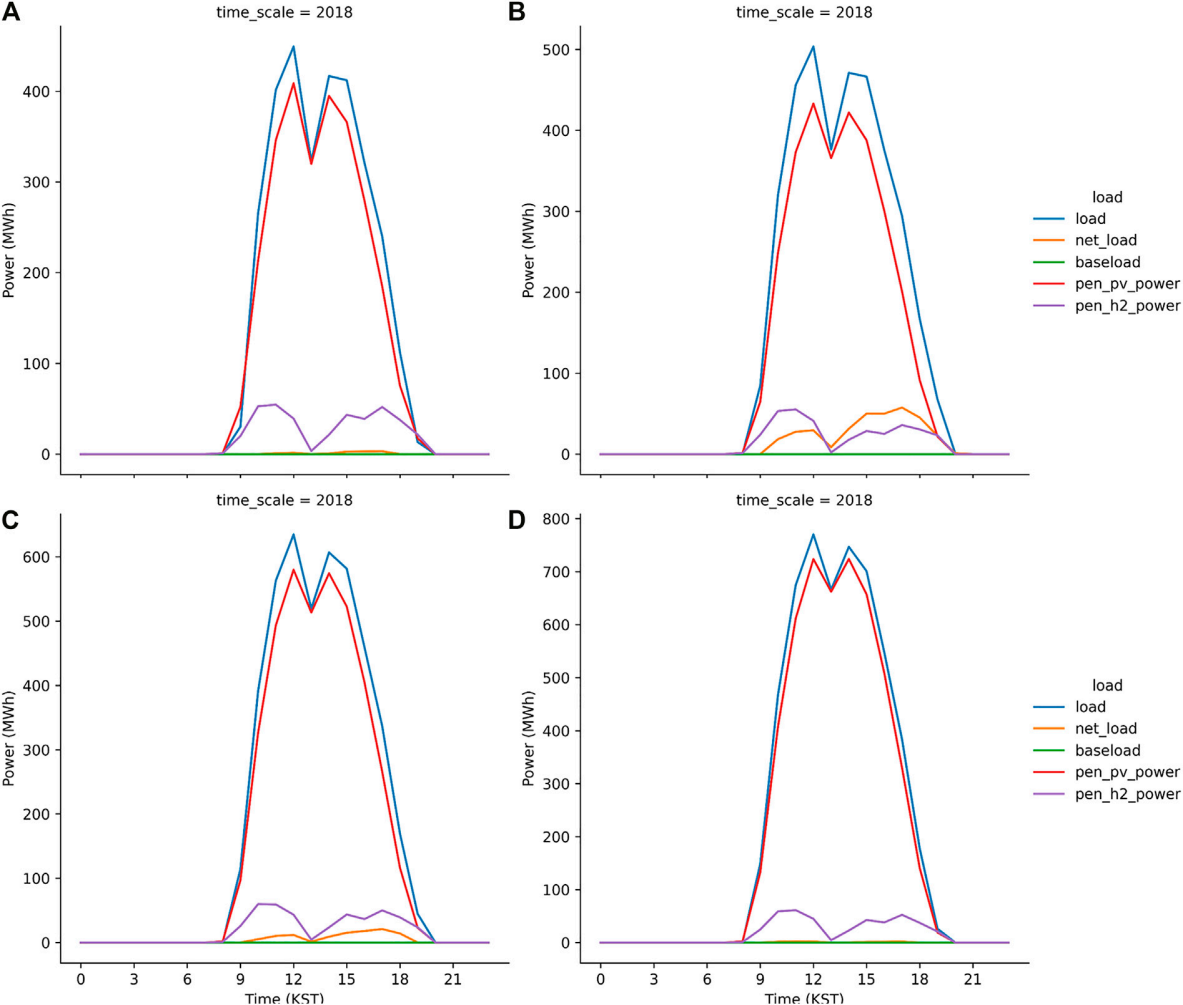
Same as Figure 8 but from S4 run **(A)**, S5 run **(B)**, S6 run **(C)** and S7 run **(D)**.

Meanwhile, the S2 run was successful because the 700 MW_p_ PV plants installed in the JN division can mitigate the peak load during daytime (Figure 11A). In contrast, the net load shown in Figure 11B is the direct result from the S3 run. It implies that the S3 run failed to operate the grid system when the peak load level is reduced to the lowest value, which is 15% of the daytime load. In other words, the primary power supplied by the solar power generation with the PEM system as the secondary source cannot supply sufficient electricity to offset the net load. We increased the PV capacity in the JN division from 700 MW_p_ to 1050 MW_p_ in the S4 run in which the level for peak load shave is identical to the S3 run. Figure 12A illustrates no net load in the S4 run, implying that the daytime load is compensated by the solar power generation and the reserved power, i.e., the PEM system with H_2_ gas. In the S5 run, which was a more aggressive strategy for the peak load shave model, the PV capacity is not sufficient to generate electricity for the load and electrolysis (Figure 12B). Moreover, the S6 run, in which the PV capacity was twice the currently installed capacity, also failed to compensate the net load (Figure 12C). Subsequently, a successful simulation S7 was performed in which the PV capacity is set extremely high to 1750 MW_p_. The results from the S7 run indicates that the peak load can be mitigated to its lowest value (10% of the daytime load) by using the solar power generation and PEM system.

Finally, the capacity factors for each simulation were compared. The annual capacity factor is calculated as follows:

$$CF\;\left(\%\right)=\frac{AEP\;\left(Wh\right)}{Capacity\;\left(W\right)\times 8760\;\left(h\right)}\times 100,$$ (8)

where AEP means the annual energy production, i.e., the solar power generation in a single year. Because the capacity factor of the PEM system cannot be calculated using Eq. 8, this study defines the operation rate as the ratio of the PEM system usage hours to 8,760 h. Table 3 summarizes the results of all the simulations. In the baseload run, the capacity factor of the PV plants is 17.5% of which 14.1% is transmitted into the electrolysis system. The PV plants must be installed to facilitate electrolysis that is coupled to the PEM system as a reserve power source. The capacity factor of the PV plants is same for all the peak load shave runs because the incoming solar irradiance is identical despite the change in PV capacity. When compared to the S2 run, in S1 run a significant portion of electricity generated by the PV plants is utilized to produce the H2 gas. However, it does not mean that the electricity is generated by the PEM system using H2 gas. The PEM usage rate is 11.2% which is much lower than 14.8% from the S2 run. The S1 run produces H_2_ gas but most of it is stored in the gas tank. As the level for the peak load shave is lowered, the capacity factor for the PV use increased. This is because the primary source for the peak load shave model is the PV plants. The simulations in the S2 and S4 runs were successful because H_2_ gas remains nominal. The PEM usage rate is more than 14.0% for these two simulations with similar capacity factors as the PV power plants. Therefore, the electrolysis–PEM system plays a significant role as the reserve power in the solar power generation during daytime.

**TABLE 3:** The summary of capacity factor, PEM usage rate and H_2_ storage resulted from the all simulations. Total, Grid and H_2_ indicate that the electricity generated by PV plants, transmitted into the grid (load) and electrolysis, respectively.

Simulation	Total (%)	Grid (%)	H_2_ (%)	PEM (%)	Storage (kg)
B1	17.5	3.4	14.1	50	-
S1	15.0	7.7	7.3	11.2	3,296
S2	15.0	8.9	6.1	14.8	697
S3	15.0	10.3	4.7	14.2	0
S4	15.0	10.5	4.5	15.4	382
S5	15.0	11.5	3.5	13.7	9
S6	15.0	11.6	3.4	15.1	64
S7	15.0	11.7	3.3	14.5	1,265

## Conclusion

This study developed a PV–electrolysis–PEM hybrid model for a feasibility study and carried out simulations for several scenarios in Korea. The UASIBS–KIER model derives the solar irradiance using satellite imagery at 1 km × 1 km pixels every 15 min over the Korean peninsula. The annual total irradiance, averaged over 24 years from 1996 to 2019 is 1,310 kWh m^−2^. In 2017, the average solar irradiance is the maximum. The spatial distribution of solar irradiance represents that the JN division out of the 17 administrative divisions in Korea is an appropriate region for solar power generation, which is consistent with the installed capacity in this region. The electricity load or demand in the JN division exhibits the peak load during daytime. At night, the demand gradually decreases and therefore, the minimum value is exhibited at four KST.

Even if the PV system was simultaneously connected to the grid and electrolysis system in the performance model, the top priority of the PV system is to supply electricity to the grid or load. Subsequently, the surplus solar power is employed to produce H_2_ gas using the electrolysis system. H_2_ gas stored in the tank can be transported into the PEM system when the solar irradiance is significantly less to generate electricity or when the electricity demand is extremely high. Solar irradiance is first converted into the POA irradiance at the azimuth and slope angles of 180° and latitude, respectively. The PV power is then estimated using Eq. 1. This study ignored the impacts of soiling and cell temperature on the efficiency of the PV module because the exact information for each module installed in the JN division is unavailable.

In this study, eight scenarios including one baseload and seven peak load shave runs were considered. The baseload run examined if the PV–electrolysis–PEM system can supply electricity for the baseload like the conventional power plants. The performance model results indicate that the capacity of the PV plants must be increased to 58.5 GW at a given baseload of 4200 MW every hour. This proves that the PV–electrolysis–PEM system is not appropriate as an alternative to the conventional power plants. Instead, this system is more efficient for the peak load shave. Subsequent simulations considering the peak load shave exhibits that the peak load can be reduced by the solar power generation and PEM system. The PEM system is more useful in winter because the solar irradiance is relatively weak in that season.

Thus, the PV–electrolysis–PEM system was investigated for various scenarios in Korea. The proposed model is simple but the first attempt to analyze the impact of electrolysis–PEM on the grid connected system. This study examined the performance model at macroscale because the electricity load information is not available in detail. Renewable energy is more efficient in a distributed power system which is directly related to the micro-grid system. The performance model gives a significant insight to develop the control logic model for the PV–electrolysis–PEM system in a micro-grid setup. In spite of elaborated effort on the performance model, the model results suggest that the H_2_ storage system is occasionally competitive to the other energy storage systems, i.e., electricity storage system as well as pumped storage. Therefore the economic analysis including the Levelized Cost of Energy still remains for the further analysis.

## Data Availability

The raw data supporting the conclusions of this article will be made available by the authors, without undue reservation.
